# Serum lipids in Brazilian children and adolescents: determining their reference intervals

**DOI:** 10.1186/s12889-015-1359-4

**Published:** 2015-01-21

**Authors:** Natasha Slhessarenko, Cristina MA Jacob, Raymundo S Azevedo, Cor JF Fontes, Glaucia V Novak, Adagmar Andriolo

**Affiliations:** Julio Müller School Hospital, Universidade Federal de Mato Grosso, Cuiabá, MT Brazil; School of Medicine, Universidade de São Paulo, São Paulo, Brazil; Department of Medicine, Universidade Federal de São Paulo, São Paulo, Brazil

**Keywords:** Serum lipids, Reference intervals, Child, Adolescent

## Abstract

**Background:**

Demographic, geographic, environmental and genetic factors influence lipids. In many countries, the normal lipid ranges for laboratory tests are based on references from American children and adolescents. In this work, we determined the reference intervals (RIs) for total cholesterol (TC), high-density lipoprotein cholesterol (HDL-c), non-high-density lipoprotein cholesterol (nHDL-c), low-density lipoprotein cholesterol (LDL-c) and triglycerides (TG) in Brazilian healthy children and adolescents.

**Methods:**

A cross-sectional study was conducted of 1,866 randomly sampled healthy children and adolescents from kindergartens and schools. Blood samples were collected after a variable period of fasting based on the age of the participant. The upper cut-off points were the 75^th^ and 95^th^ percentiles for TC, nHDL-c, LDL-c and TG. The 10^th^ percentile (low) was used as the bottom level for HDL-c. Non-parametric tests were used for statistical analyses.

**Results:**

The following RI and 75^th^ and 95^th^ percentiles were observed for each age interval. The 95^th^ percentile values obtained for TC were: 1 to 2 years, 189 mg/dL, 3 to 8 years, 199 mg/dL; 9 to 12 years, 205 mg/dL. For the nHDL c, the only age group 1 to 12 years, this percentile value was 150 mg/dL. For the LDL-cholesterol, the values corresponding to the percentiles above, aged 1 to 8 years and 9 to 12 years, were 132 mg/dL 139 mg/dL, respectively. For the triglycerides, the values corresponding to 95^th^ percentile were: 1 year, 189 mg/dL; 2 to 5 years, 139 mg/dL; 6 to 12 years, 139 mg/dL . The 10^th^ percentiles for HDL-c were 24 mg/dL, 28 mg/dL, 32 mg/dL and 36 mg/dL for children 1, 2, 3 and 4-12 years old, respectively.

**Conclusions:**

The lipid reference intervals defined in the studied Brazilian children and adolescents differ from those recommended by the international literature and should be used for clinical decisions contributing to improve the diagnosis in this particular group in our country.

## Background

Cardiovascular disease is a leading cause of death in many countries, and epidemiological studies have linked this disease to elevations in serum lipids, such as total cholesterol (TC), non-high-density lipoprotein cholesterol (nHDL-c), low-density lipoprotein cholesterol (LDL-c) and triglycerides (TG), and a low concentration of high-density lipoprotein cholesterol (HDL-c); together, these changes contribute to the development of atherosclerosis [1-6]. The onset of this disease occurs during the first years of life, and coupled with the increased prevalence of dyslipidemia in the pediatric group, the evaluation of serum concentrations of these parameters at early ages is of utmost importance [5-9].

The definition of reference intervals (RIs) poses an arduous challenge for clinical laboratories worldwide, and the difficulties are even greater in the pediatric population [10-12]. Ideally, laboratories should define RIs based on their own populations [10]. This practice allows for accurate clinical interpretation and decision making. However, most laboratories adopt the values reported by the diagnostic test manufacturer or the medical literature [13,14]. Currently, a world task force involving several centers worldwide is striving to establish pediatric RIs [15-18].

The RIs for parameters such as serum lipids have been established using national and international consensuses that defined decision limits [10]. The decision limits specified in the medical literature are based on proposals of the National Cholesterol Education Program (NCEP) in 1992 and were updated by the National Heart, Lung and Blood Institute (NHLBI) in 2012 [1,5]. These guidelines are based on studies conducted over more than 3 decades. There are three guidelines on lipids and lipoprotein for children and adolescents in Brazil, including the 1^st^ Brazilian Guideline for the Prevention of Atherosclerosis in Childhood and Adolescence [19], the 5^th^ Brazilian Guideline on Dyslipidemia and the Prevention of Atherosclerosis [20] and the 1^st^ Brazilian Guideline on Familial Hypercholesterolemia [21]. However, these guidelines use the RIs, not for their own population, but for American children and adolescents. This fact can lead to misinterpretation because demographic characteristics and variables, such as diet and geographic regions, may interfere with these parameters [1,5,10]. Therefore, lipid parameters for the local population determined by local studies with well-defined protocols are essential for the correct interpretation of laboratory tests and clinical decision making. This study determined the RIs for TC, HDL-c, n-HDLc, LDL-c and TG in healthy children and adolescents from Cuiabá City in Midwest Brazil.

## Methods

### Demographic and geographic data

**Figure 1:**
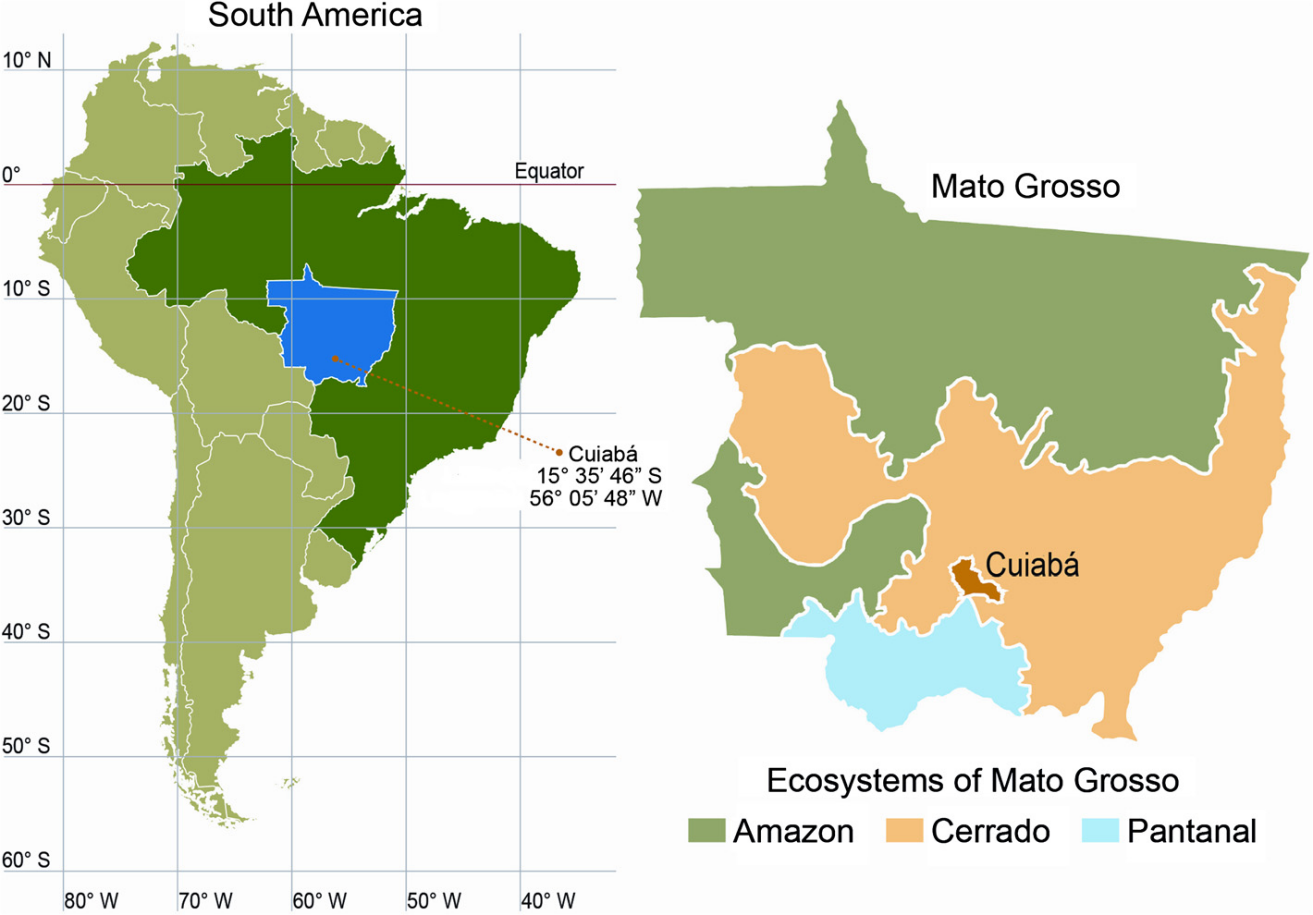
**Map of South America: Cuiabá, Mato Grosso, is located in central Brazil; the three ecosystems of the state are shown.**

The city of Cuiabá, capital of Mato Grosso, is in the central part of South America, and it has a large, diverse population living in three different ecosystems (Figure 1). The ethnic composition of the city is the result of two distinct historical phases, the first occurring during the gold rush in the 18^th^ century, with miscegenation between whites and Native Indians and later with slaves from Africa. The second phase of miscegenation occurred in the 1970s with the migration of people from southern and southeastern Brazil, consisting of people from many ethnic backgrounds, mainly of European descent. The migration was so extensive that the number of inhabitants in the capital quintupled in three decades. Therefore, the residents of this city come from various regions, which makes this city a representative place for an RI study from an ethnographic point of view.

The sample size calculation was based on CLSI recommendations, which require at least 120 reference individuals per age group [10]. This cross-sectional study evaluated 1,994 healthy children and adolescents who were randomly sampled from 20 schools and 25 daycare centers. Interviewers completed a written questionnaire assessing the individual’s background, his or her relatives and demographic and anthropometric data. The following inclusion criteria were used for this study: children and adolescents from 1 to 12 years, 11 months and 29 days of age without any underlying disease or diagnosed clinical complaints at the time of blood collection. Children and adolescents who had a chronic disease or who were taking medications that were not reported in the questionnaire but were discovered during clinical evaluation were excluded. Children and adolescents were included after parents or guardians signed the informed consent. All data were entered into Epidata 3.1 with a double entry. Of the 1,994 individuals who participated, 128 were excluded for the regular use of medication (n = 14, 12 children and 2 adolescents) or acute clinical symptoms present at the time of blood collection (n = 114, 100 children and 14 adolescents). Complaints referred to at the time of collection, which resulted in the exclusion of children, were fever (40 children and 5 adolescents), sore throat (28 children and adolescents 6), otalgia (27 children and 2 adolescents), dysuria (5 children) and dengue (1 teenager). Children taking the following medications were also excluded: Carbamazepine (4 children), Phenobarbital (3 children), Ritalin (1 child and 1 teenager), Seretide (2 children), Sabril (1 child), prophylactic Benzetacil (1 teenager) and tuberculosis prophylaxis (1 child).

The blood samples were collected after a fasting period of 3 hours for children from 1 to 2 years old, 6 hours for children from 2 to 5 years old and 12-14 hours for older children and adolescents. The samples were processed in a Cobas® 6000 analyzer (Roche Hitachi Cobas 6000 Analyzer, Hitachi High Technologies Corp., Tokyo, Japan) using enzymatic colorimetric and enzymatic homogeneous colorimetric methods. The nHDL-c and LDL-c levels were calculated (nHDL-c = TC - HDL-c and LDL-c = TC – HDL-c – TG/5), and the latter parameter was calculated using the Friedwald formula.

### Ethics

The study was approved by the Ethics Committees at the Julio Müller University Hospital (# 947/2010) and the Faculty of Medicine of the Universidade de São Paulo (# 318/2011). The Municipal Department of Education and Health of Cuiabá city also reviewed and approved this study.

### Statistics

We tested the homogeneity of variances using Bartlett’s test for each parameter by age, and depending on the result, ANOVA or a Kruskal-Wallis test was used to examine differences among the age groups. The Bonferroni post-hoc test was applied to verify pairwise differences between the means by ages if the ANOVA or Kruskal-Wallis test yielded a p-value less than 0.05, and age intervals with similar means were combined. Bartlett’s test was applied to the new age groups, followed by ANOVA or a Kruskal-Wallis test to check whether the new age groups maintained the age categorization. We excluded outliers (±3 standard deviations from the mean), and the RI was established as the resulting mean ±2 standard deviations. In addition, we calculated the percentile distribution to establish the RIs and adopted the criteria of the NHLBI Expert Panel on Integrated Guidelines for Cardiovascular Health and Risk Reduction in Children and Adolescents [5] as a proposed decision limit for this population. For TC, nHDL-c, LDL-c and TG, values below the 75^th^ percentile were considered desirable or acceptable. Values from the 75^th^ to approximately the 95^th^ percentile were considered borderline, and values greater than or equal to the 95^th^ percentile were considered high. For HDL-c, the 10^th^ percentile was used as the lower limit. Therefore, the participants below the 10^th^ percentile were considered to have a low concentration. A concentration above the 50^th^ percentile was considered desirable. The significance level was 5% for all tests. The statistical analyses were performed using Minitab software, version 15 (Minitab, PA, USA) and SPSS, version 16 (Chicago, Illinois, USA).

## Results

**Figure 2:**
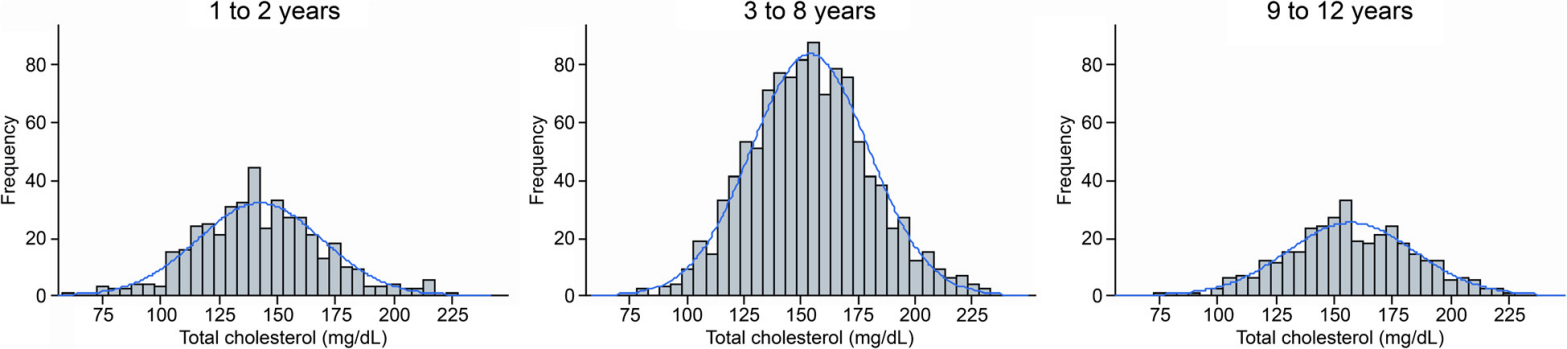
**Histograms by age group for total cholesterol (mg/dL), after excluding outliers, with coupled Gaussian curves.**

**Figure 3:**
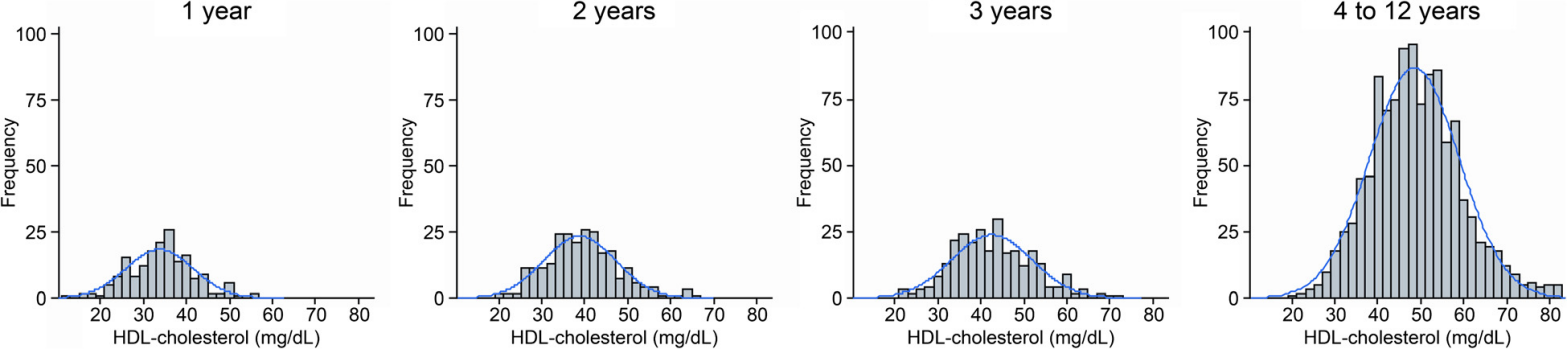
**Histograms by age group for HDL cholesterol (mg/dL), after excluding outliers, with coupled Gaussian curves.**

**Figure 4:**
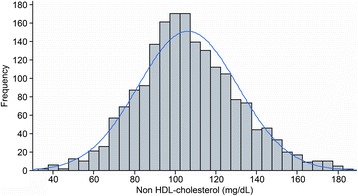
**Histograms by age group for nHDL cholesterol (mg/dL), after excluding outliers, with coupled Gaussian curves.**

**Figure 5:**
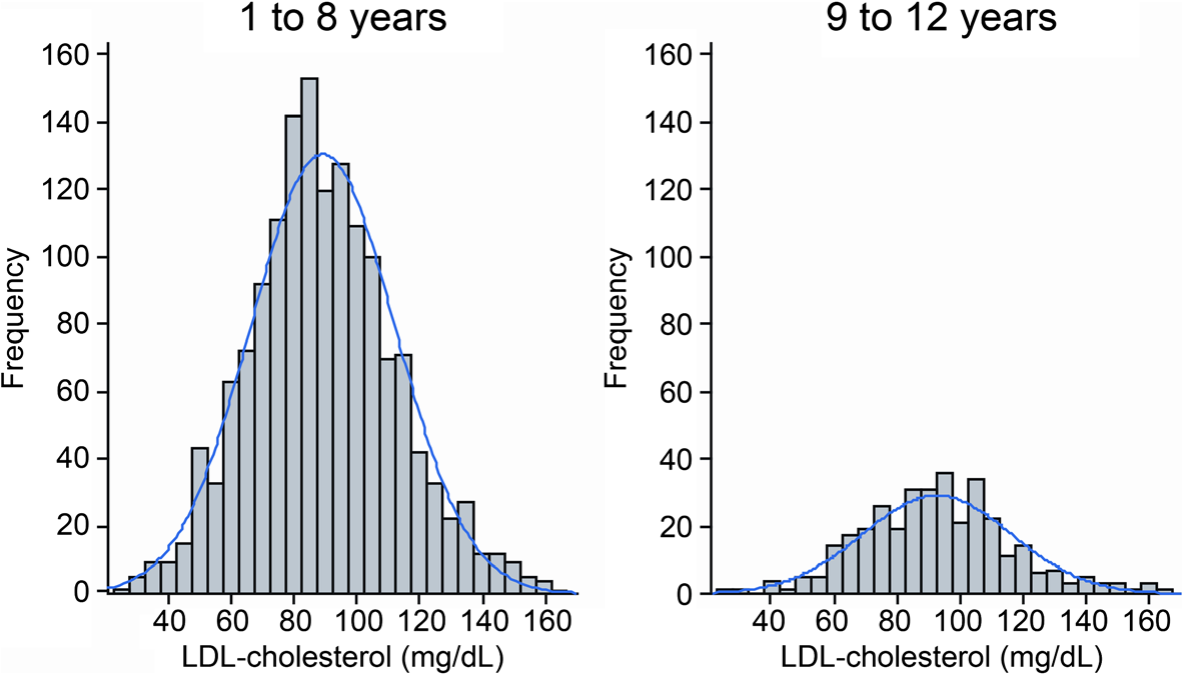
**Histograms by age group for LDL cholesterol (mg/dL), after excluding outliers, and coupled Gaussian curves.**

**Figure 6:**
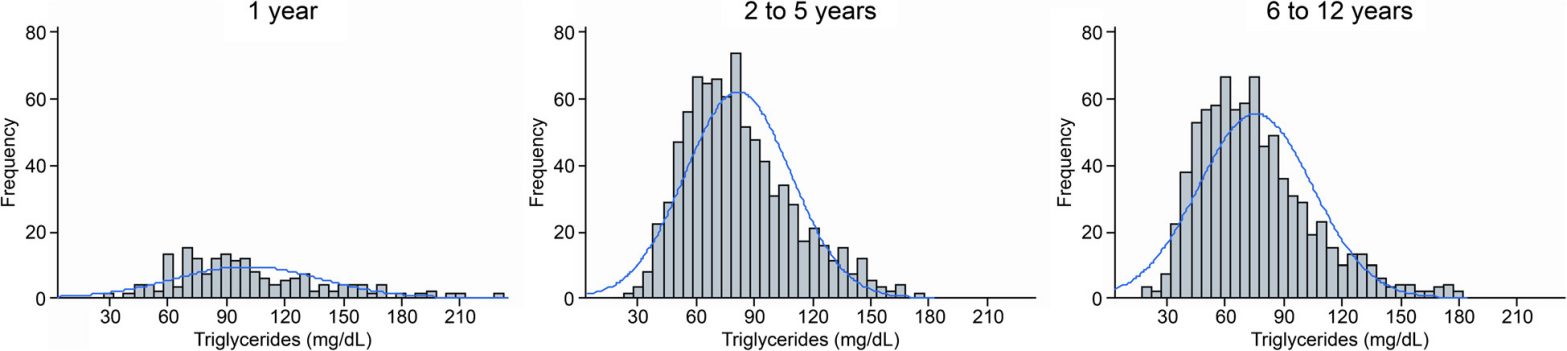
**Histograms by age group for triglycerides (mg/dL), after excluding outliers, with coupled Gaussian curves.**

The final study population consisted of 1,866 healthy children and adolescents, and 919 (49.2%) were male. Regarding race, 64.4% were declared as mixed race, 19.6% as white, 12.5% as black and 2.8% as Asian descendent. A total of 97% were born in Cuiabá. Their nutritional statuses were classified as 73.4% normal weight, 9.4% overweight, 6.3% at risk of being overweight, 6.9% obese, 2.7% underweight and 1.2% extremely underweight.^26^ This classification was performed using WHO Anthros and WHO Anthros Plus version 3.2.2. A total of 39.2% of all participants had a family history of diabetes, and 65.5% and 22.4% reported a family history of hypertension and obesity, respectively. Cardiovascular diseases, such as myocardial infarction and stroke, were reported by 17.6% and 12.3%, respectively. Frequency histograms of the serum levels of TC (Figure 2), HDL-c (Figure 3), non-HDL-c (Figure 4), LDL-c (Figure 5) and triglycerides (Figure 6) for all age analyzed for all groups after excluding the outliers are shown in Figures 2, 3, 4, 5, and 6 respectively.

The RIs for TC were calculated for 3 age groups: 1-2 years, 3-8 years and 9-12 years. Four age groups were calculated for HDL-c: 1 year, 2 years, 3 years and 4-12 years. All children and adolescents were grouped into a single age group for nHDL-c. Two age groups were defined for LDL-c (1-8 years and 9-12 years), and 3 age groups were used for TG (1 year, 2-5 years and 6-12 years). The proposed RIs (±2 SD) for TC were 89-196 mg/dL for 1-2-year-olds, 102-205 mg/dL for 3-8-year-olds and 103-212 mg/dL for 9-12-year-olds. The proposed RIs (±2 SD) for HDL-c were 18-49 mg/dL for 1-year-olds, 22-55 mg/dL for 2-year-olds, 24-61 mg/dL for 3-year-olds and 27-69 mg/dL for 4-12-year-olds. The proposed RI (±2 SD) for nHDL-c was 57-155 mg/dL for 1-12-year-olds. The proposed RIs (±2 SD) for LDL-c were 43-135 mg/dL for 1-8-year-olds and 45-140 mg/dL for 9-12-year-olds. The proposed RIs (±2 SD) for TG were 26-176 mg/dL for 1-year-olds, 27-135 mg/dL for 2-5-year-olds and 17-134 mg/dL for 6-12-year-olds. The mean, standard deviation, maximum and minimum and 1^st^ and 3^rd^ quartiles values of each parameter evaluated in their respective age groups are shown in Table 1. Many researchers have emphasized the need to consider gender in the determination of the RI for laboratory parameters for the pediatric population. Statistical significance in this study was observed for some parameters in specific age groups, but these differences would not likely have a relevant clinical impact (Table 2).

**Table 1:** **Estimated parameters for the sample distribution of children and adolescents included in the study to establish the reference intervals for lipids after excluding the outliers**

**Parameter**	**Age group (years)**	**n**	**Mean (SD)**	**1** ^**st**^ **quartile**	**Median**	**3** ^**rd**^ **quartile**	**Range (mg/dL)**	**RI (mg/dL)**
Total cholesterol (mg/dL) n = 1858	1 to 2	428	142.6 (26.7)	125	141	159.8	62 - 225	89-196
	3 to 8	1,085	153.4 (25.9)	136	153	169	79 - 231	102-205
	9 to 12	345	157.2 (27.3)	139	155	176	74 - 244	103-212
HDL cholesterol (mg/dL) n = 1848	1	179	33.8 (7.8)	29	34	39	12 - 56	18-49
	2	245	38.8 (8.4)	33	39	44	19 - 65	22-55
	3	279	42.6 (9.3)	36	42	49	22 - 71	24-61
	4 to 12	1,145	48.5 (10.5)	41	48	55	20 - 82	27-69
Non-HDL cholesterol (mg/dL) n = 1854	1 to 12	1,854	106.4 (24,5)	90	104.5	122	33 - 183	57-155
LDL cholesterol (mg/dL) n = 1856	1 to 8	1,512	89.3 (23.1)	74	88	103	24 - 161	43-135
	9 to 12	344	92.6 (23.5)	76.2	92	106	25 - 167	45-140
Triglycerides (mg/dL) n = 1839	1	178	100.8 (37.4)	73	93	123.8	31 - 230	26-176
	2 to 5	847	81.2 (27.1)	61	77	97	25 - 175	27-135
	6 to 12	814	75.7 (29.1)	54	71	90	22 - 181	17-134

SD: standard deviation; RI: reference interval.

**Table 2:** **Statistical significance observed for the HDL-cholesterol (HDL-c), non-HDL-cholesterol (n-HDL-c), LDL-cholesterol (LDL-c) and triglyceride (TG) parameters in specific age groups by gender**

**Parameter**	**Age group**	**Male**	**Female**	**p value**
**(mg/dL)**	**(years)**	**(mean)**	**(mean)**	
HDL-c	2	40	37.7	0.026
	4 – 12	49.3	47.7	0.011
n-HDL-c	1 – 12	103.9	108.7	0.001
LDL-c	1– 8	87.5	91.1	0.002
TG	2 – 5	79.1	83.2	0.027
	6 – 12	70.7	80.4	0.001

The following 75^th^ and 95^th^ percentiles were observed for each age interval. The values obtained for TC were: 1 to 2 years, 160 mg/dL and 189 mg/dL; 3 to 8 years, 170 mg/dL and 199 mg/dL; 9 to 12 years, 176 mg/dL and 205 mg/dL, respectively. For the nHDL c, the only age group 1 to 12 years, this percentiles values were 122 mg/dL and 150 mg/dL, respectively. For the LDL-cholesterol, the values corresponding to the percentiles above, aged 1 to 8 years and 9 to 12 years, were 104 mg/dL and 132 mg/dL; 106 mg/dL and 139 mg/dL, respectively. For the triglycerides, the values corresponding to these percentiles were: 1 year, 127 mg/dL and 189 mg/dL; 2 to 5 years, 98 to 139 mg/dL; 6 to 12 years, 92 mg/dL and 139 mg/dL. The 10^th^ percentiles for HDL-c were 24 mg/dL, 28 mg/dL, 32 mg/dL and 36 mg/dL for children 1, 2, 3 and 4-12 years old, respectively. The clinical decision limits for each parameter, calculated according to the NHLBI,^5^ are shown in Tables 3 and 4.

**Table 3:** **Desirable, borderline–high and high serum lipid concentrations (mg/dL) for children and adolescents from Cuiabá, Brazil**

**Parameter**	**Age group**	**Desirable**	**Borderline-High**	**High**
**(mg/dL)**	**(years)**	**<P75**	**P75 - P95**	**≥P95**
Total cholesterol	1 – 2	<160	160-188	≥189
	3 – 8	<170	170-198	≥199
	9 – 12	<176	176-204	≥205
Non-HDL cholesterol	1 – 12	<122	122-149	≥150
LDL cholesterol	1 – 8	<104	104-131	≥132
	9 – 12	<106	106-138	≥139
Triglycerides	1	<127	127-188	≥189
	2 – 5	<98	98-138	≥139
	6 – 12	<92	92-138	≥139

**Table 4:** **Desirable, borderline and low serum HDL-cholesterol concentrations (mg/dL) for children and adolescents from Cuiabá, Brazil**

**Parameter**	**Age group**	**Desirable**	**Borderline-high**	**Low**
**(mg/dL)**	**(years)**	**>P50**	**P10 - P50**	**<P10**
HDL cholesterol	1	>34	25 – 34	24
	2	>39	29 – 39	28
	3	>42	33 – 42	32
	4 - 12	>48	37 – 48	36

## Discussion

This study is the first performed in Brazil in a large population that included children of early ages from the city of Cuiabá, which is located in a central area of South America. This city presents very interesting peculiarities for study due to the ethnic mixture and an intense migratory movement of people from different regions of Brazil with different eating habits and genetic backgrounds.

Cardiovascular diseases usually manifest after the fourth decade of life, but atherosclerosis begins during an individual’s first few years [1,5,6]. This finding emphasizes the need to determine the real concentrations of lipids in children and adolescents and identify individuals with cardiovascular risk indicators to enable early intervention.

The values used to diagnose dyslipidemia vary among populations. Each country should ideally establish its own RIs [10,22]. The reference values for lipids are probably not country-specifics. However, it is believed that race, lifestyle, diet, environment and economic development among different areas could account for the differences in lipid levels in children of different countries [23].

Some studies on lipid RIs have been conducted in Brazil, but the goals were to determine the distribution of serum lipids in pediatric populations [24-27], except one study in a small town in São Paulo [28,29]. This report is the first Brazilian investigation to establish the RIs for serum lipids in children and adolescents aged 1–12 years. The currently recommended lipid RIs for Brazilian children and adolescents are based on references from American children and adolescents, who have different genetic profiles and distinct dietary habits. Therefore, this study aimed to narrow this gap. The current cohort is a specific Brazilian population, presumably with non-ideal demographic characteristics, but the results of this study are very interesting. The above-mentioned population has more characteristics in common with Brazilian children and adolescents from other regions of the country than with any North American cohort.

The NHLBI [5] combined children and adolescents 2–19 years of age in a single group to assess their TC, HDL-c, nHDL-c and LDL levels. Only two age groups, 0–10 years old and 10–19 years old, were used to assess the TG levels. In the CALIPER study [30] and the present study, however, the parameters were studied in different age groups for each analyte.

According to the NHLBI, a TC level <170 mg/dL is desirable in children and adolescents 2–19 years of age [5]. The desirable borderline and upper limit threshold values obtained in this study were lower in the age group of 1–2 years. The desirable borderline and upper concentrations for the age group of 3–8 years overlap the recommendations of the NHLBI. Higher thresholds were obtained in the group of 9–12 years than the thresholds proposed by the American guidelines (Table 3).

The CALIPER study [30] combined children and adolescents into age groups of 0–14 days, 15 days to <1 year and 1–19 years. Our results were compared only with this last age group. We obtained lower upper limit values for children up to 8 years of age and values above the CALIPER guidelines for children and adolescents aged >9 years. Genetic differences and diverse eating habits among the Brazilian, US and Canadian populations are possible factors for the observed differences. Compared with the previous Brazilian study of lipids in a single age group (2-9 years old), our values were identical (i.e., the desirable limit for this analyte was 170 mg/dL) for 3-8 year olds [28].

An HDL-c level >45 mg/dL is desirable, 40–45 mg/dL is borderline, and <40 mg/dL is low according to the NHLBI. This last value represents approximately the 10^th^ percentile, and it is the most relevant value in medical practice [5]. The values corresponding to desirable, borderline and lower limits in this study are below the limits suggested by the NHLBI for the age groups of 1 year, 2 years and 3 years (Table 4). There are two potential reasons for this difference: the concentration of this analyte increases gradually over time, and this parameter was studied in a single age group (2–19 years) in the NHLBI study. The concentrations were higher in the age group 4–12 years, and although they were borderline and within desirable limits, they were below the threshold compared with the values proposed by the NHLBI (Table 4). The CALIPER study defined the following four age groups for HDL-c: 15 days to <1 year, 1 to <4 years, 4 to <13 years and 13 to <19 years. The lower and upper limits obtained in the present study were lower than the CALIPER concentrations in the corresponding age groups [30]. Possible explanations include the genetic differences and diverse eating habits of the two analyzed populations. The previous Brazilian study of lipids in children does not reference the 10^th^ percentile, but the desirable limit includes greater values than the results of our study [28].

The n-HDL-c value is obtained by subtracting the HDL-c value from the TC value. Acquiring this value does not require fasting, which renders it very attractive, especially among children. Moreover, this parameter is predictive of atherosclerosis because it includes all classes of lipoproteins [5,31]. The NHLBI considers concentrations < 120 mg/dL desirable [5]. Our values are higher than those suggested by NHLBI in the three categories, namely, desirable, borderline and upper limit (Table 3). The lower HDL-c concentrations in this population (Tables 3 and 4) may explain this difference. The value for this parameter in our study was greater than that in the previous Brazilian lipid study [28]. The CALIPER study did not report this parameter in their analysis [30].

A comparison between the concentrations of LDL-c obtained in this study and the NHLBI revealed that the LDL-c 75^th^ percentile in our study was lower than the NHLBI desirable limit. The LDL-c concentrations of the borderline upper limit for the 1-8-year-old age group overlapped with the NHLBI recommendations. The concentration obtained for this parameter in the age group 9–12 years was higher than the recommendations of the NHLBI (Table 3). One possible explanation for this discrepancy may be methodological differences because the NHLBI data were clustered into a single age group. Differences in the TC, HDL-c and TG values may contribute to these disparities. The value for this parameter in our study was higher than that in the previous Brazilian study of lipids [28]. The CALIPER study did not report this parameter in their analysis [30].

The TG thresholds in this study were systematically higher than those proposed by the NHLBI (Table 3) [5]. The fasting period may provide one explanation for this effect. The recommended fasting for the collection of this analyte is 12–14 hours, but the recommended fasting intervals (duration between feedings) in this study were 3 hours for children up to 2 years, 6 hours for children up to 5 years and 12-14 hours for children > 5 years.

The TG levels obtained in this study [30] show that the upper and lower limits were systematically lower compared with CALIPER. The fasting time may be one explanation for this effect because fasting was not required in the CALIPER study. The value for this parameter in our study was greater than that in the previous Brazilian study of lipids [28].

The weakness of this study was in not includind children from different places around the country. However, the studied children are descended from parents from various regions of Brazil and have many characteristics in common with other Brazilian children and adolescents. In addition, not including adolescents older than 13 years old and the long duration of fasting in younger children are other concerns. New studies need to fully fill this gap and include older age groups in this analysis. Further studies of the serum lipid concentrations and clinical and laboratory parameters already evaluated in this work in our population will be performed in future analyses.

## Conclusions

In conclusion, this study found that the distribution curves of the lipids and lipoprotein concentrations differed among children of different ages in Brazil, which suggests the importance of considering the age partition in developing lipid guidelines. Obtaining more discriminatory data allows the identification of more sensitive cut-offs, which are useful in guiding clinical decisions. Moreover, the use of lipid reference ranges for children and adolescents who have been exposed to the same demographic and geographic conditions may identify alterations more effectively.

## Abbreviations

CALIPER: Canadian laboratories initiative on pediatric reference intervals
CLSI: Clinical and laboratory standards institute
HDL-c: HDL cholesterol
LDL-c: LDL cholesterol
nHDL-c: Non-HDL cholesterol
NCEP: National cholesterol education program
NHLBI: National heart, lung and blood institute
RI: Reference intervals
TC: Total cholesterol
TG: Triglycerides
